# Computed tomographic imaging features to differentiate gastric schwannomas from gastrointestinal stromal tumours: a matched case–control study

**DOI:** 10.1038/s41598-023-43902-4

**Published:** 2023-10-16

**Authors:** Lijia Wang, Qi Wang, Li Yang, Chongfei Ma, Gaofeng Shi

**Affiliations:** Department of Radiology, The Fourth Hospital of Hebei Medical University and Hebei Provincial Tumor Hospital, 12 Jiangkang Road, Shijiazhuang, 050011 Hebei Province China

**Keywords:** Diseases, Gastroenterology, Medical research, Oncology

## Abstract

To investigate clinical data and computed tomographic (CT) imaging features in differentiating gastric schwannomas (GSs) from gastric stromal tumours (GISTs) in matched patients, 31 patients with GSs were matched with 62 patients with GISTs (1:2) in sex, age, and tumour site. The clinical and imaging data were analysed. A significant (*P* < 0.05) difference was found in the tumour margin, enhancement pattern, growth pattern, and LD values between the 31 patients with GSs and 62 matched patients with GISTs. The GS lesions were mostly (93.5%) well defined while only 61.3% GIST lesions were well defined.The GS lesions were significantly (*P* = 0.036) smaller than the GIST lesions, with the LD ranging 1.5–7.4 (mean 3.67 cm) cm for the GSs and 1.0–15.30 (mean 5.09) cm for GIST lesions. The GS lesions were more significantly (*P* = 0.001) homogeneously enhanced (83.9% vs. 41.9%) than the GIST lesions. The GS lesions were mainly of the mixed growth pattern both within and outside the gastric wall (74.2% vs. 22.6%, *P* < 0.05) compared with that of GISTs. No metastasis or invasion of adjacent organs was present in any of the GS lesions, however, 1.6% of GISTs experienced metastasis and 3.2% of GISTs presented with invasion of adjacent organs. Heterogeneous enhancement and mixed growth pattern were two significant (*P* < 0.05) independent factors for distinguishing GS from GIST lesions. In conclusion: GS and GIST lesions may have significantly different features for differentiation in lesion margin, heterogeneous enhancement, mixed growth pattern, and longest lesion diameter, especially heterogeneous enhancement and mixed growth pattern.

## Introduction

Gastric subepithelial tumours are not rare even though there is no definitive epidemiology of these tumours, which may cover a variety of pathological types, including benign or malignant tumours, a tumour-like lesion or a tumour with malignant potential. Gastrointestinal stromal tumours (GISTs) have the highest incidence and the most significant biological diversity in this large group of tumours. Other tumours or tumour-like lesions similar to GISTs include leiomyoma, gastric schwannoma (GS), neurofibroma, glomus tumour, and inflammatory fibroid polyp of the stomach and ectopic pancreas^1,2^. Among them, leiomyomas tend to occur around the cardia and gastric fundus, and glomus tumours often have significant specific enhancement on imaging examination. The incidence of neurofibroma is very low, and they are mostly reported in case reports. More than 75% of inflammatory fibroid polyps of the stomach manifest as nodules growing in the cavity of the gastric antrum^3^. In typical ectopic pancreas whose onset site is close to the pylorus, the imaging enhancement characteristics are similar to those of the pancreas and are characterized by intraluminal growth and an oval-shaped mass with a long axis along the gastric wall^4^. GS is a relatively rare submucosal tumour derived from the gastrointestinal tract and occurs mostly in the stomach, accounting for 2%-6% of all gastrointestinal mesenchymal tumours^5–7^. Its histological and imaging features are quite different from those of soft tissues or central nervous system tumours^8–10^. As a type of mass with a relatively homogeneous texture, smooth edges but no capsules^11^, the lesions are mostly benign and slow-growing. GS almost never relapses after surgery^12^, and endoscopic resection of the tumour can achieve satisfactory therapeutic effects^13^, which is significantly different from the treatment strategy and prognosis of GISTs^14^. If GSs can be distinguished from GISTs by imaging features, this will greatly help with the later treatment of the patients. Some studies^5,6^ have shown that the imaging features of enhanced computed tomography (CT) scans and tumour morphology can be used to distinguish GSs from GISTs. However, the imaging features of GSs overlap much with those of GISTs, which is one of the challenges in distinguishing the two. It was hypothesized that a combination of clinical characteristics and CT imaging features of GSs could be used to differentiate GSs from GISTs. This study was consequently performed to investigate the clinical characteristics and CT imaging features of GSs in distinguishing the two diseases using matched cases for control.

## Patients and methods

### Patients

**Figure 1:**
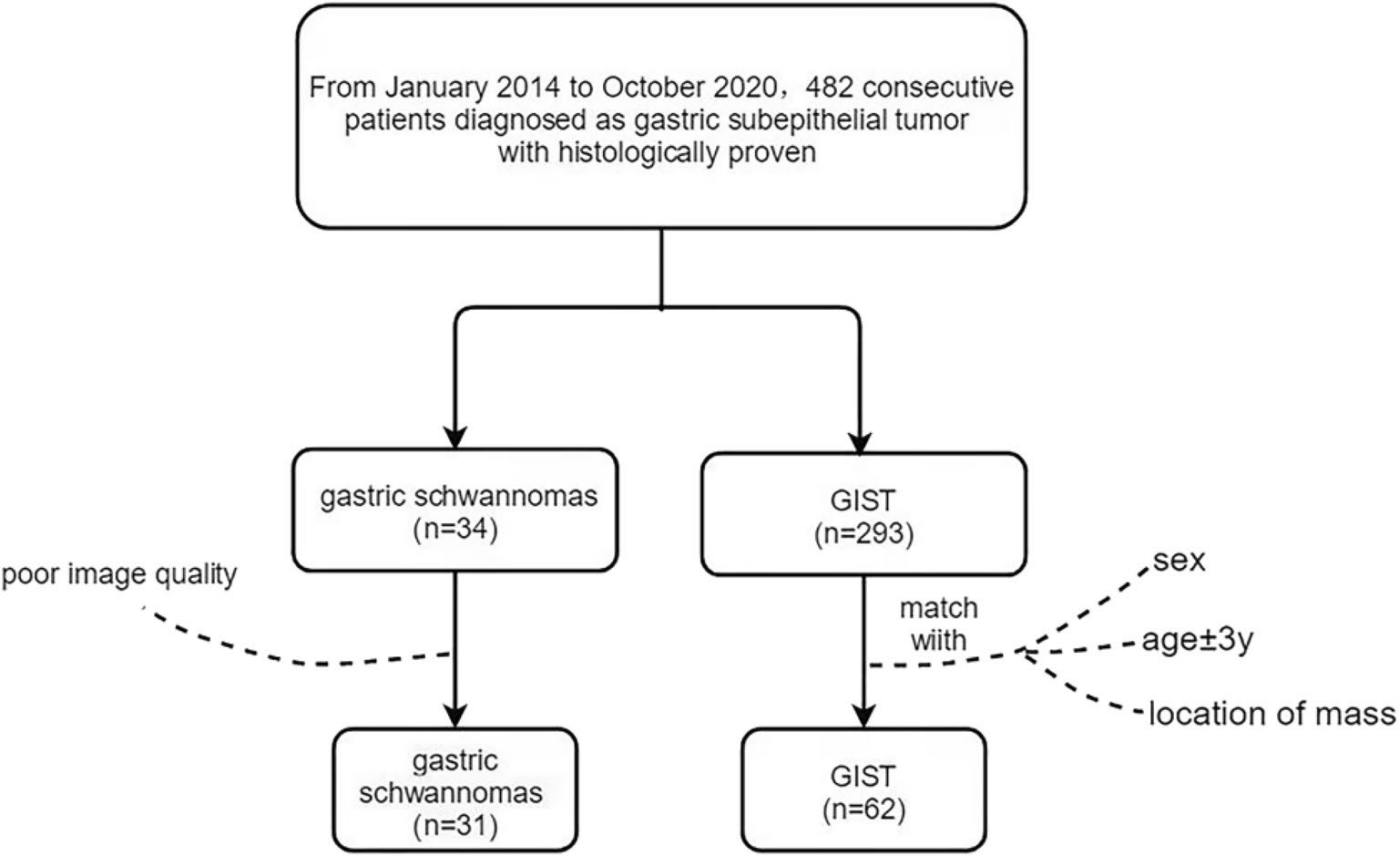
Flowchart showing the inclusion process based on number ratio of 1:2 for matching the control. GISTs, gastrointestinal stromal tumors.

This retrospective one-center study was approved by the ethics committee of Hebei Medical University Affiliated Forth Hospital, and the informed consent was waived by the same ethics committee of the Fourth Hospital of Hebei Medical University because of the retrospective study design. All methods were conducted in accordance with the relevant guidelines and regulations. From January 2014 to October 2020, a total of 482 consecutive patients with gastric subepithelial tumours confirmed by surgical pathology and immunohistochemical examinations were identified from the picture archiving and communication (PACS) database in our hospital. Figure 1 shows the sample selection procedure, in which 293 cases with GISTs and 34 patients with GSs were identified. The inclusion criteria were consecutive patients with GSs or GISTs, clear multiphase enhanced scanning images of the upper abdomen with multislice spiral CT and 1–1.5-mm thin-slice images, and largest diameter of the lesions greater than 1 cm. Patients with poor imaging who did not meet the above criteria were excluded.

### CT examinations and data collection

The medical information of the patients, including clinical data, surgical findings, and results of pathologic examination, was collected. Abdominal contrast-enhanced CT examinations were performed using a multidetector row CT (MDCT) scanner: a Brilliance IC (Philips, Best, The Netherlands) and SOMATOM Definition Flash (Siemens Healthcare, Erlangen, Germany) CT: a Lightspeed Pro 32 (GE Healthcare, Chicago, IL). Patients were instructed to fast for at least 6 h before CT scanning. Ten minutes before the scan, each patient was administered 10 mg of anisodamine intramuscularly to reduce peristalsis of the gastrointestinal tract before drinking 500–1000 mL water to expand the stomach fully. For MDCT scanners, the scanning parameters were as follows: detector configuration 0.625–1 mm, pitch 0.891–1.35; rotation time 0.5–0.75 s, 120kVp, and 150–250 mAs. MDCT images were reconstructed with a slice thickness and reconstruction interval of 3 mm. For 3D reconstruction, MDCT images were reconstructed with a slice thickness and reconstruction interval of 1–1.5 mm. In addition to axial MDCT images, coronal and sagittal multiplanar reformatted images were captured.

### Image analysis

The image features of gastric tumours (Table 1) were derived from the medical record by two radiologists who were blinded to the patient data. Disagreement in extracting the imaging features of morphological pattern, feature and degree of contrast enhancement of the tumours was solved by discussion or involvement of a senior radiologist with clinical experience of over 10 years. The attenuation of each lesion, the longest diameter (LD), the vertical diameter (VD), and the LD/VD ratio were measured. The contours of tumours were classified into round-like and irregular shapes, with Dumbbell-shaped and lobulated masses being assigned to irregular shapes. The edges were divided into well-defined and ill-defined ones. Presence or absence of mucosal ulceration on the surface of the tumour was recorded. No ring-shaped enhancement pattern was present. The enhancement mode was divided into homogeneous and heterogeneous enhancement, and the growth pattern into endoluminal, exoluminal, or mixed^15^. To measure the maximal enhancement degree of the lesion, the lesion was measured in the area with the most significant enhancement in the venous phase, judged by the naked eye. In the arterial phase, the same site and the same size region of interest were selected as in the venous phase. When measuring, blood vessels visible to the naked eye and necrotic areas were avoided. The degree of enhancement was divided into good (degree of enhancement over 40 HU), moderate (degree of enhancement ranging 20–40 HU), and poor (degree of enhancement less than 20 HU). For tumour growth, lymph nodes surrounding the lesion with a short diameter of more than 5 mm were defined as positive. Tumour invasion of surrounding organs or distant metastasis was also recorded. Tumour location was defined as follows: 1, the gastric fundus and cardia; 2, gastric body; 3, gastric antrum.

**Table 1:** Data in GSs and GISTs before and after matching.

Characteristics	Before matching		*P* value	After matching		*P* value
	GSs (31)	GISTs (213)		GSs (31)	GISTs (62)	
Age, median (range)	56(36–77)	58(29–83)	0.333	56(36–77)	57(39–77)	0.689
Man sex, n(%)	9(29)	114(53.5)	0.012	9(29)	18(29)	1
Location			0.000			1
Gastric fundus and cardia	3(9.7)	114(53.5)		3(9.7)	6(9.7)	
Gastric body	26(83.9)	78(26.6)		26(83.9)	52(83.9)	
Gastric antrum	2(6.5)	21(9.9)		2(6.5)	4(6.5)	
Contour			0.013			0.079*
Round-like	29(93.5)	157(73.7)		29(93.5)	48(77.4)	
Irregular	2(6.5)	56(26.3)		2(6.5)	14(22.6)	
Margin			0.001			0.004*
Well defined	28(90.3)	127(59.6)		28(90.3)	38(61.3)	
Ill defined	3(9.7)	86(40.4)		3(9.7)	24(38.8)	
Surface			0.049			0.242
Regular	24(77.4)	124(58.2)		24(77.4)	40(64.5)	
Mucosal ulceration	7(22.6)	89(41.8)		7(22.6)	22(35.5)	
Enhancement pattern			0.001			0.001*
Homogeneous	26(83.9)	99(46.5)		26(83.9)	26(41.9)	
Heterogeneous	5(16.1)	114(53.5)		5(16.1)	36(58.1)	
Enhancement grades in AP			0.221			0.191*
Good	0(0)	19(8.9)		0(0)	6(9.7)	
Moderate	10(32.3)	65(30.5)		10(32.3)	20(32.3)	
Poor	21(67.7)	129(60.6)		21(67.7)	36(58.1)	
Enhancement grades in VP			0.258			0.317
Good	12(38.7)	58(27.2)		12(38.7)	26(41.9)	
Moderate	17(54.8)	123(57.7)		17(54.8)	26(41.9)	
Poor	2(6.5)	32(15)		2(6.5)	10(16.1)	
Growth pattern			0.001			0.001*
Endoluminal	2(6.5)	104(48.8)		2(6.5)	23(37.1)	
Exoluminal	6(15.4)	52(24.4)		6(15.4)	25(40.3)	
Mixed	23(74.2)	57(26.8)		23(74.2)	14(22.6)	
LD (cm)	3.67 ± 1.55	5.47 ± 3.69	0.013	3.67 ± 1.55	5.09 ± 3.14	0.036*
LD/VD ratio	1.26 ± 0.17	1.34 ± 0.27	0.29	1.26 ± 0.17	1.35 ± 0.32	0.427
Hemorrhage	0(0)	5(2.3)	1	0(0)	1(1.6)	1
Calcification	4(12.9)	43(20.2)	0.466	4(12.9)	17(27.4)	0.187*
Perilesional LNs	0(0)	5(2.8)	1	0(0)	0(0)	
Metastasis	0(0)	10(4.7)	0.37	0(0)	1(1.6)	0.551
Invasion of adjacent organs	0(0)	9(4.2)	0.608	0(0)	2(3.2)	

Data were presented in frequency and percentages in parentheses. *P* values were obtained with Fisher’s exact test and Mann–Whitney U test. *Included in the multiple logistic regression models (*P* < 0.2). GS, gastric schwannomas; GIST, gastric stromal tumour; AP, Arterial phase; VP, Venous phase; LNs, lymph nodes; LD, the longest diameter; VD, the vertical diameter.

### Statistical analysis

The statistical analysis was performed with the SPSS version 25 (SPSS, IBM, Chicago, IL, USA). We used a 1:2 matching ratio for selection of the control group to reduce selections bias. To evaluate the differences in patient characteristics, including age, sex, and tumour-specific data, between the two groups, the Mann–Whitney U test, Pearson’s chi-squared test, and Fisher’ exact test were used. Variables with *P* < 0.2 in the bivariate analysis were included into the multivariate regression models. Conditional logistic regression analysis was used to estimate the odds ratios (ORs) and 95% confidence intervals (CIs). The receiver operating characteristics (ROC) curve analysis was performed to investigate the diagnostic ability of relevant parameters. In all tests, a value of p˂0.05 was considered a significant difference.

### Ethical approval

This retrospective one-center study was approved by the ethics committee of Hebei Medical University Affiliated Forth Hospital, and the informed consent was waived by the same ethics committee because of the retrospective study design.

## Results

Totally, 31 patients with GSs (aged 56 years in mean and 36–77 years in range) and 213 cases with GISTs (aged 58 years in mean and 36–77 years in range) were identified to meet the inclusion criteria (Table 1). The ratios of males to females among GS and GIST patients were 1:2.44 and 1.15:1, respectively, and the sex distribution of the GIST group was fairly even. The majority of GSs were located in the gastric body (including the gastric angle) (83.9%). In contrast, GISTs originated mainly in the gastric fundus and cardia (53.5%).

The GS group consisted of 9 men aged 62.5 (rang 50–77) years and 22 women aged 54.9 (range 36–77) years. A group of GISTs patients were randomly selected as the control group to match the GS group (n = 31) in the following parameters: 1:2 in number, sex, age ± 3 years, and location of the tumour (Table 2). Sixty-two patients with GISTs who matched the GS patients were enrolled for analysis.

**Table 2:** Univariable and multivariable analysis.

Variable	Univariable analysis			Multivariable analysis		
	Hazard ratio	95%CI	*P* value	Hazard Ratio	95%CI	*P* value
Irregular contour	0.332	0.079–1.391	0.053			
Ill defined margin	0.262	0.08–0.861	0.004			
Heterogeneous	0.244	0.094–0.635	0.001	0.300	0.114–0.792	0.015
Poor enhancement grades in arterial phase	0.677	0.351–1.307	0.154			
Mixed growth pattern	2.95	1.602–5.432	0.000	2.498	1.393–4.477	0.002
The longest diameter, LD	0.840	0.701–1.006	0.036			
Calcification	0.508	0.178–1.452	0.116			

*CI* confidence interval; *LD* the longest diameter.

Before matching, a significant (*P* < 0.05) difference existed in the sex component, tumour location, tumour contour, margin, surface, enhancement pattern, growth pattern, and LD values between 31 patients with GSs and 213 patients with GISTs (Table 1).

**Figure 2:**
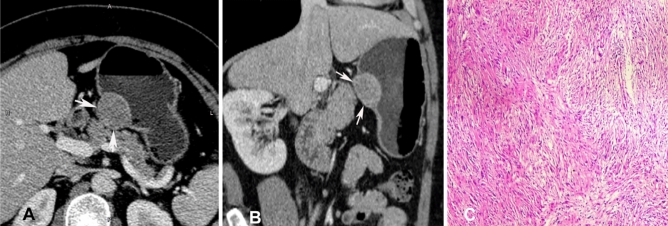
Gastric Schwannoma at the gastric body in a 51-year-old woman. A&B. Transverse (**A**) and (**B**) sagittal CT scans showed a well-defined, homogeneous ovoid mass (arrows) with mixed growth pattern in the gastric body. (**C**) The surgical resection histopathology was schwannoma with a size of 3.25 cm, which was composed of spindle cells (hematoxylin–eosin staining, × 10). Immunohistochemical staining indicated CD117( −), DOG-1( −), Des ( −), Actin ( −), Ki67(7%), S100( +), Vimentin ( +), and SDHB ( +).

**Figure 3:**
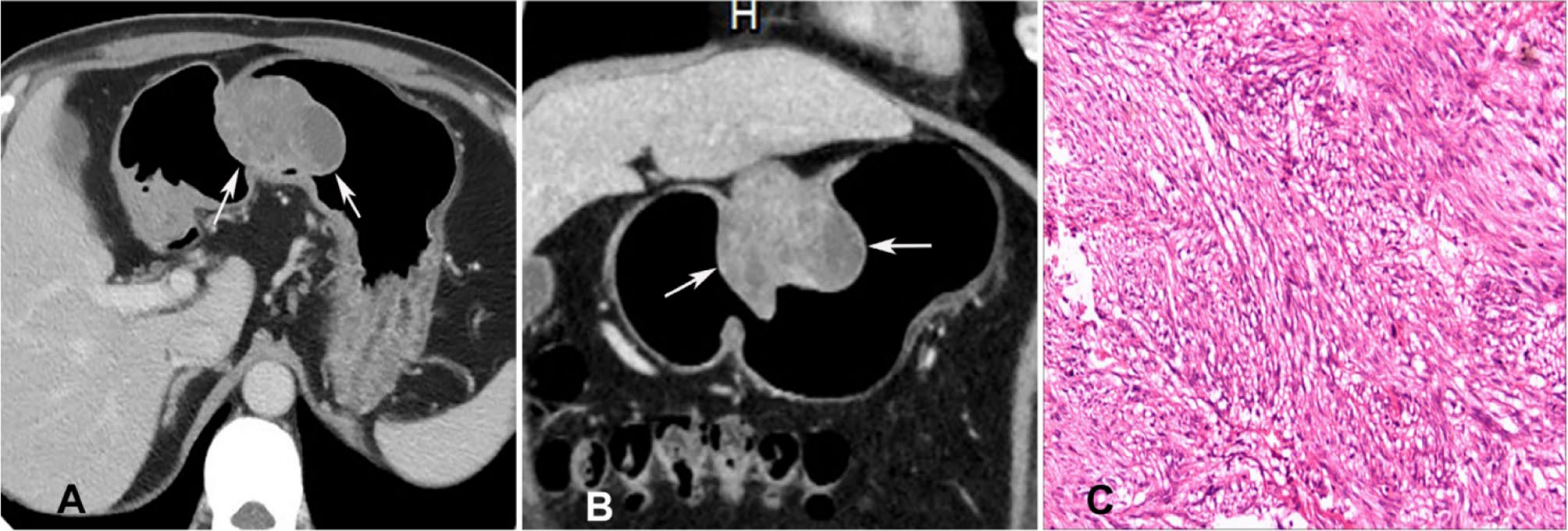
A gastrointestinal stromal tumor (GIST) was shown at the gastric body in a 56-year-old man. A&B. Transverse (**A**) and (**B**) coronal contrast-enhanced CT scans showed a well-defined, heterogeneously low-attenuating irregular mass (arrows). The overlying mucosa (arrow) suggested a submucosal lesion. (**C**) The histopathological result of surgical resection demonstrated a GIST lesion with a size of 6.36 cm, which was composed of spindle cells (hematoxylin–eosin staining, × 10). Immunohistochemical staining revealed CD34( +), CD117( +), DOG-1( +), Des ( −), Actin ( −), Ki67(7%), S100( −), and Vimentin ( +).

After matching with the 31 patients with GSs in the age, sex and tumour location, a significant (*P* < 0.05) difference was still found in the tumour margin, enhancement pattern, growth pattern, and LD values between the 31 patients with GSs and 62 matched patients with GISTs (Table 2). In lesion margin (Figs. 2 and 3), the GS lesions were mostly (93.5%) well defined while only 61.3% GIST lesions were well defined. In LD values, the GS lesions were significantly (*P* = 0.036) smaller than the GIST lesions, with the LD ranging 1.5–7.4 (mean 3.67 cm) cm for the GSs and 1.0–15.30 (mean 5.09) cm for GIST lesions. The LD/VD ratio was not significantly (*P* > 0.05) different between the two groups. In contrast enhancement, the GS lesions were more significantly (*P* = 0.001) homogeneously enhanced (83.9% vs. 41.9%) than the GIST lesions even though the enhancement degree in the arterial phase or venous phase was not significantly different (*P* = 0.191 and 0.317, respectively). The growth pattern of tumour was significantly (*P* < 0.05) different between the two lesions, with the GS lesions mainly of the mixed growth pattern both within and outside the gastric wall (74.2% vs. 22.6%). No significant (*P* > 0.05) differences were detected in tumour contour, presence of mucosal ulceration in the gastric cavity, haemorrhage, calcification, perilesional lymph nodes, metastasis, and invasion of adjacent organs (Table 2). No metastasis or invasion of adjacent organs was present in any of the GS lesions, however, 1.6% of GISTs experienced metastasis and 3.2% of GISTs presented with invasion of adjacent organs.

In univariate analysis of risk factors for different lesions, ill-defined lesion margin, heterogeneous enhancement, mixed growth pattern, and longest lesion diameter were significant (*P* < 0.05) features for differentiating GS from GIST lesions. In mutlvariate analysis, heterogeneous enhancement and mixed growth pattern were two significant (*P* < 0.05) independent factors for distinguishing GS from GIST lesions.

**Figure 4:**
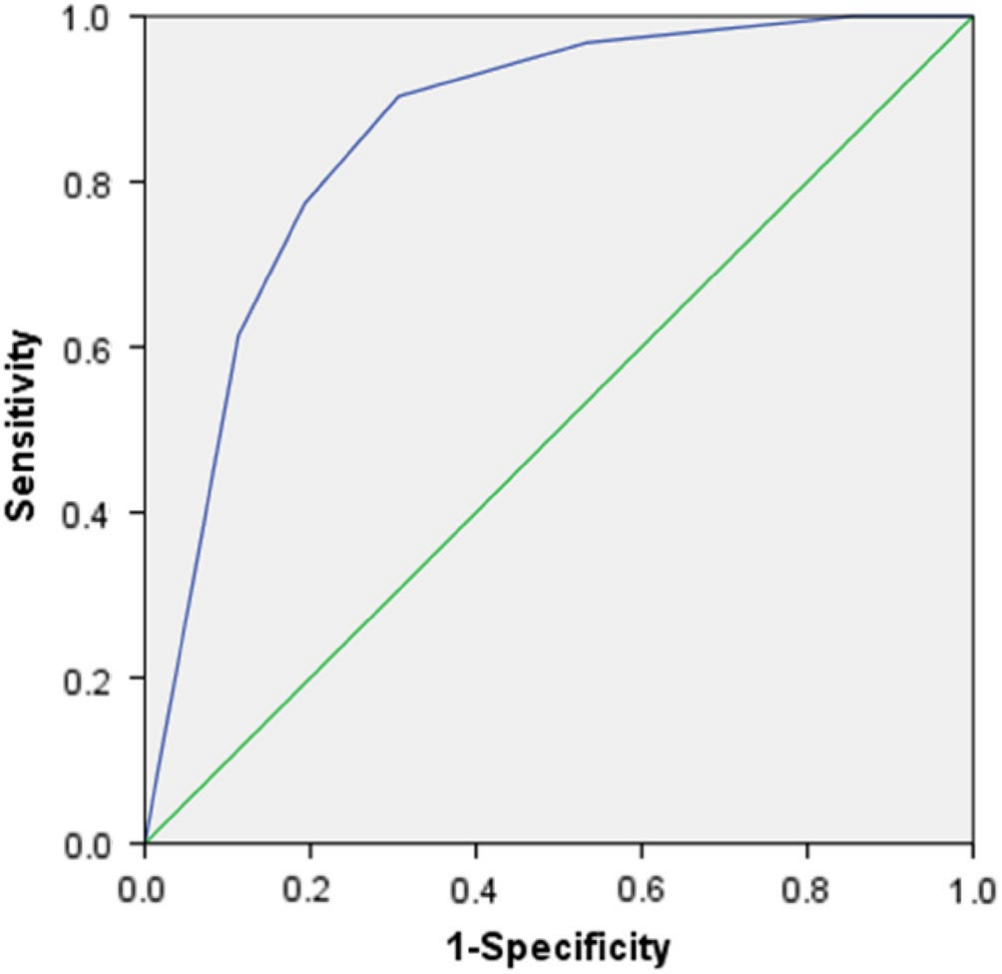
A joint receiver operating characteristic (ROC) curve analysis was performed on the two indicators of whether the tumor was homogeneous after enhancement and tumor growth pattern, with a significant (*P* < 0.001) AUC (95% CI) 0.859 (0.782–0.936), indicating a better joint diagnostic efficacy. The sensitivity of the joint diagnosis was 0.903, the specificity was 0.694, and the Youden index was 0.597.

**Figure 5:**
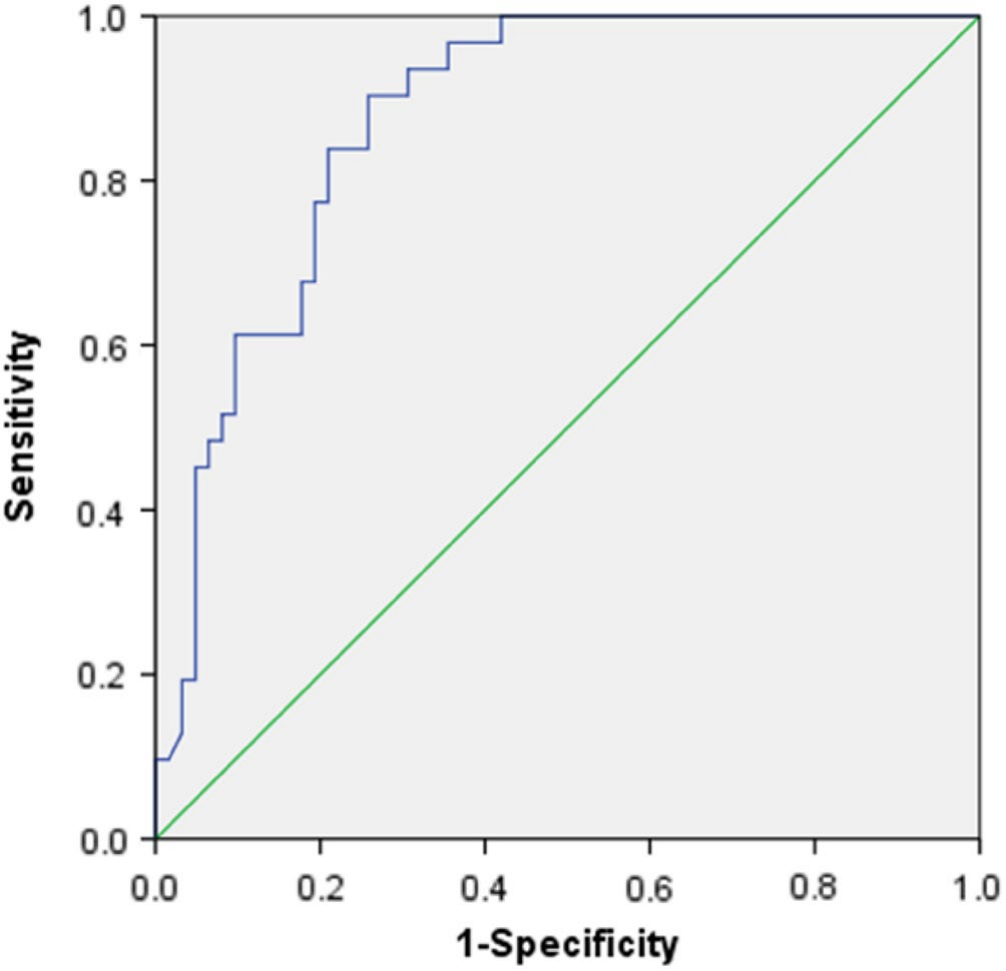
A receiver operating characteristic (ROC) curve analysis was conducted jointly on four indicators: the maximal diameter of the tumor, edge of the tumor, whether the tumor was homogeneous after enhancement, and the tumor growth pattern, resulting in a significant (*P* < 0.001) joint AUC (95% CI) 0.875 (0.807–0.944) and an optimal joint diagnostic performance. The sensitivity of the joint diagnosis was 0.903, the specificity was 0.742, and the Youden index was 0.645.

The four meaningful parameters obtained in the univariate analysis, including the maximal diameter of the tumor, edge of the tumor, whether the tumor was homogeneous after enhancement, and tumor growth pattern, were individually and jointly analyzed by the ROC curve analysis (Table 3). The combined diagnosis of the four indicators showed good accuracy, with an area under the ROC curve (AUC) of 0.875 (0.807–0.944). The combination of two indicators of whether the tumor was homogeneous after enhancement and tumor growth pattern also achieved satisfactory results of an AUC 0.859 (0.782–0.936) (Table 3, Figs. 4, 5).

**Table 3:** Receiver operating characteristic analysis.

Variable	AUC (95% CI)	*P* value	Sensitivity	Specificity	Youden index
① LD	0.366 (0.253–0.480)	0.036	0.871	0.161	0.032
② Margin	0.355 (0.242–0.468)	0.023	0.097	0.613	− 0.290
③ Enhancement pattern	0.290 (0.182–0.399)	0.001	0.161	0.419	− 0.420
④ Growth pattern	0.219 (0.120–0.318)	< 0.001	0.065	0.629	− 0.306
③ ④	0.859 (0.782–0.936)	< 0.001	0.903	0.694	0.597
①②④④	0.875 (0.807–0.944)	< 0.001	0.903	0.742	0.645

The receiver operating characteristic (ROC) curve analysis was performed separately on the four indicators of the maximal diameter of the tumor, edge of the tumor, whether the tumor was homogeneous after enhancement, and tumor growth pattern. The results showed that the diagnostic prediction was statistically significant (*P* < 0.05), but both of the areas under the curve (AUC) were less than 0.5, indicating that the individual diagnostic performance of these four indicators was poor. A joint ROC curve analysis was performed on the two indicators of whether the tumor was homogeneous after enhancement and tumor growth pattern. The joint diagnostic efficacy was better. A ROC curve analysis was conducted jointly on four indicators: the maximal diameter of the tumor, edge of the tumor, whether the tumor was homogeneous after enhancement, and tumor growth pattern. The joint diagnostic performance was optimal.

## Discussion

In this study investigating the value of the clinical data and CT imaging features in differentiating GSs from GISTs in matched patients, it was found that GS and GIST lesions may have significantly different features for differentiation in lesion margin, heterogeneous enhancement, mixed growth pattern, and longest lesion diameter, especially heterogeneous enhancement and mixed growth pattern. ROC curve analysis revealed that joint diagnosis of the latter two indicators can achieve good prediction results (with an AUC of 0.859). This is similar to the combined diagnostic value using all four indicators (with an AUC of 0.875). This study may suggest that even if gastric stromal tumors and gastric schwannoma have similar onset locations, some reference indicators exist that can provide valuable identification information to distinguish the two tumors.

GSs are a relatively rare benign gastric subepithelial tumour that often occurs in the gastric body^7–10,16^. Before matching in this study, 83.9% of GSs occurred in the gastric body, whereas only 26.6% of GISTs occurred in the gastric body. Because of the overlapping in the location of disease, it is difficult to distinguish the two types of tumour by location alone. Therefore, the onset location was treated as one of the factors for case matching. The incidence of GSs in females was also higher than that in males in our study, consistent with the findings by Choi et al.^17^. Because of the similar incidence of GISTs in females and males, sex was also used as one of the matching factors. A 1:2 matching ratio was used to match the GSs and GISTs patients based on age, sex, and tumour location to explore the value of tumour imaging characteristics in the differentiating GSs and GISTs.

The two most valuable indicators in differentiating the two tumours were the growth and enhancement pattern. Among the GSs, 74.2% (23/31) showed bidirectional growth across the gastric wall, whereas only 22.6% (14/62) of GISTs had this growth pattern. GSs rarely had an exogenous growth pattern on the gastric wall (6.5%, 2/31), whereas GISTs had a significantly greater proportion in the three growth modes. In enhancement pattern, 83.9% (26/31) of GS cases was relatively homogeneous after enhancement, showing progressive enhancement, whereas only 41.9% (26/62) GISTs exhibited this pattern.

Lin et al.^6^ found that GSs were different from schwannomas in other organs which were more homogeneous on CT images before and after enhancement. In the study by Wang et al.^18^, 18 of 19 GSs patients (94.7%) showed homogeneous enhancement. Therefore, homogeneous enhancement pattern was typical of GSs. Heterogeneous enhancement of GISTs is more common, consistent with the heterogeneity of these tumours. Although the growth and enhancement patterns were two significant independent factors for distinguishing the two tumours, they were not sufficient because of overlapping of these features in some tumours.

Our results showed that the LD of GSs was in the range of 1.5 cm to 7.4 cm, in line with the findings by Mekras et al.^7^. Overall, GSs are relatively small. This is consistent with the slow growth of benign tumours. Irregular shape and large tumour diameter are more common in GISTs. With the characteristics of tumour enlargement, fast and outward growth, an irregular shape of the tumour is bound to appear besides the feature of a large lesion. However, we did not find a value of the optimal diameter in distinguishing the two types of tumours. Choi et al.^17^ found that the GS lesion size of smaller than 5 cm might be used to distinguish from GISTs, but their cohort was too small besides some confounding factors.

In both types of tumours, progressive enhancement was the dominant form of CT enhancement, with similar enhancement pattern and degree. When the tumour grows into the gastric cavity, ulcers may occur on the surface of the tumour, which is the reason why patients with either tumour may have gastrointestinal bleeding^7,19^. Both tumours have the possibility of calcification, but intratumoural bleeding is rare. Only five GISTs showed intratumoural bleeding in this study. Metastasis and surrounding tissue invasion were also only seen in patients with GISTs. These features are external manifestations of the malignancy of GISTs and highly suggested that GISTs are malignant^14,20^.

In our study, no enlargement of peripheral lymph nodes was observed in cases of gastric schwannoma which is a benign tumor of the stomach. Even if some researchers have discovered the presence of lymph nodes in gastric schwannomas, it may be just an accidental phenomenon rather than a must because of the benign nature of gastric schwannoma.

Some limitations existed in this study, including the retrospective and once-center study design, a small cohort of patients, Chinese patients enrolled only, and further investigation using radiomics^21^ like others did to differentiate GSs from GISTs, which may all affect the outcome of this study. Future prospective, randomized, multi-center studies involving multiple races and ethnicities will have to be performed to reach more valuable and better outcomes.

In conclusion, GS and GIST lesions may have significantly different features for differentiation in lesion margin, heterogeneous enhancement, mixed growth pattern, and longest lesion diameter, especially heterogeneous enhancement and mixed growth pattern.

## Data Availability

The data can be obtained from the corresponding author on reasonable account.

## Abbreviations

CT: Computed tomography
GS: Gastric schwannoma
GIST: Gastric stromal tumour
LD: Longest diameter
AP: Arterial phase
VP: Venous phase
VA: Vertical diameter
LN: Lymph node
CI: Confidence interval

## References

[1] Gong J, Kang W, Zhu J, Xu J (2012). CT and MR imaging of gastrointestinal stromal tumor of stomach: A pictorial review. Quant. Imaging Med. Surg..

[2] Xing JJ, Huang WP, Wang F, Chai YR, Gao JB (2022). Computed tomography features and clinicopathological characteristics of gastric glomus tumor. BMC Gastroenterol..

[3] Kang HC, Menias CO, Gaballah AH (2013). Beyond the GIST: Mesenchymal tumors of the stomach. Radiographics..

[4] Rezvani M, Menias C, Sandrasegaran K, Olpin JD, Elsayes KM, Shaaban AM (2017). Heterotopic pancreas: histopathologic features, imaging findings, and complications. Radiographics..

[5] Li R, Gan H, Ni S, Fu Y, Zhu H, Peng W (2019). Differentiation of gastric schwannoma from gastric gastrointestinal stromal tumor with dual-phase contrast-enhanced computed tomography. J. Comput. Assist. Tomogr..

[6] Liu J, Chai Y, Zhou J, Dong C, Zhang W, Liu B (2017). Spectral computed tomography imaging of gastric schwannoma and gastric stromal tumor. J. Comput. Assist. Tomogr..

[7] Mekras A, Krenn V, Perrakis A (2018). Gastrointestinal schwannomas: A rare but important differential diagnosis of mesenchymal tumors of gastrointestinal tract. BMC Surg..

[8] Levy AD, Quiles AM, Miettinen M, Sobin LH (2005). Gastrointestinal schwannomas: CT features with clinicopathologic correlation. AJR Am. J. Roentgenol..

[9] Lin YM, Chiu NC, Li AF, Liu CA, Chou YH, Chiou YY (2017). Unusual gastric tumors and tumor-like lesions: Radiological with pathological correlation and literature review. World J. Gastroenterol..

[10] Voltaggio L, Murray R, Lasota J, Miettinen M (2012). Gastric schwannoma: A clinicopathologic study of 51 cases and critical review of the literature. Hum. Pathol..

[11] Ji JS, Lu CY, Mao WB, Wang ZF, Xu M (2015). Gastric schwannoma: CT findings and clinicopathologic correlation. Abdom. Imaging.

[12] Hong X, Wu W, Wang M, Liao Q, Zhao Y (2015). Benign gastric schwannoma: How long should we follow up to monitor the recurrence? A case report and comprehensive review of literature of 137 cases. Int. Surg..

[13] Zhai YQ, Chai NL, Zhang WG (2020). Endoscopic versus surgical resection in the management of gastric schwannomas. Surg. Endosc..

[14] Parab TM, DeRogatis MJ, Boaz AM (2019). Gastrointestinal stromal tumors: a comprehensive review. J. Gastrointest. Oncol..

[15] Kim JY, Lee JM, Kim KW (2009). Ectopic pancreas: CT findings with emphasis on differentiation from small gastrointestinal stromal tumor and leiomyoma. Radiology..

[16] Richman DM, Tirumani SH, Hornick JL (2017). Beyond gastric adenocarcinoma: Multimodality assessment of common and uncommon gastric neoplasms. Abdom Radiol (NY)..

[17] Choi JW, Choi D, Kim KM (2012). Small submucosal tumors of the stomach: differentiation of gastric schwannoma from gastrointestinal stromal tumor with CT. Korean J Radiol..

[18] Wang W, Cao K, Han Y, Zhu X, Ding J, Peng W (2019). Computed tomographic characteristics of gastric schwannoma. J. Int. Med. Res..

[19] Raber MH, Ziedses des Plantes CM, Vink R, Klaase JM (2010). Gastric Schwannoma Presenting as an Incidentaloma on CT-Scan and MRI. Gastroenterol. Res..

[20] Wei SC, Xu L, Li WH (2020). Risk stratification in GIST: shape quantification with CT is a predictive factor. Eur. Radiol..

[21] Wang J, Xie Z, Zhu X (2021). Differentiation of gastric schwannomas from gastrointestinal stromal tumors by CT using machine learning. Abdom. Radiol. (NY)..

